# Association of AST/ALT ratio with 90-day outcomes in patients with acute exacerbation of chronic liver disease: a prospective multicenter cohort study in China

**DOI:** 10.3389/fmed.2024.1307901

**Published:** 2024-03-21

**Authors:** Huimin Liu, Hai Li, Guohong Deng, Xin Zheng, Yan Huang, Jinjun Chen, Zhongji Meng, Yanhang Gao, Zhiping Qian, Feng Liu, Xiaobo Lu, Yu Shi, Jia Shang, Huadong Yan, Yubao Zheng, Zixuan Shen, Liang Qiao, Weituo Zhang, Xianbo Wang

**Affiliations:** ^1^Center of Integrative Medicine, Beijing Ditan Hospital, Capital Medical University, Beijing, China; ^2^Department of Traditional Medicine, Beijing Tsinghua Changgung Hospital, School of Clinical Medicine, Tsinghua University, Beijing, China; ^3^Department of Gastroenterology, Ren Ji Hospital, School of Medicine, Shanghai Jiao Tong University, Shanghai, China; ^4^Department of Infectious Diseases, Southwest Hospital, Third Military Medical University (Army Medical University), Chongqing, China; ^5^Department of Infectious Diseases, Institute of Infection and Immunology, Union Hospital, Tongji Medical College, Huazhong University of Science and Technology, Wuhan, China; ^6^Department of Infectious Diseases, Hunan Key Laboratory of Viral Hepatitis, Xiangya Hospital, Central South University, Changsha, China; ^7^Hepatology Unit, Department of Infectious Diseases, Nanfang Hospital, Southern Medical University, Guangzhou, China; ^8^Department of Infectious Diseases, Hubei Clinical Research Center for Precise Diagnosis and Treatment of Liver Cancer, Taihe Hospital, Hubei University of Medicine, Shiyan, Hubei, China; ^9^Department of Hepatology, The First Hospital of Jilin University, Changchun, China; ^10^Department of Liver Intensive Care Unit, Shanghai Public Health Clinical Centre, Fudan University, Shanghai, China; ^11^Department of Infectious Diseases and Hepatology, The Second Hospital of Shandong University, Jinan, China; ^12^Infectious Disease Center, The First Affiliated Hospital of Xinjiang Medical University, Urumqi, China; ^13^The State Key Laboratory for Diagnosis and Treatment of Infectious Diseases, The First Affiliated Hospital of School of Medicine, Zhejiang University, Hangzhou, China; ^14^Department of Infectious Diseases, Henan Provincial People's Hospital, Zhengzhou, China; ^15^Department of Hepatology, Key Laboratory of Diagnosis and Treatment of Digestive System Tumors of Zhejiang Province, Hwamei Hospital, Ningbo No. 2 Hospital, University of Chinese Academy of Sciences, Ningbo, China; ^16^Department of Infectious Diseases, The Third Affiliated Hospital of Sun Yat-Sen University, Guangzhou, Guangdong, China; ^17^Clinical Research Center, Shanghai Jiao Tong University School of Medicine, Shanghai, China

**Keywords:** aspartate aminotransferase/alanine aminotransferase ratio, cirrhosis, advanced fibrosis, risk factor, short-term outcome, prognosis

## Abstract

**Background and aim:**

A high aspartate aminotransferase/alanine aminotransferase (AST/ALT) ratio is associated with liver injury in liver disease; however, no data exist regarding its relationship with 90-day prognosis in patients with acute exacerbation of chronic liver disease.

**Methods:**

In this study, 3,758 participants (955 with advanced fibrosis and 2,803 with cirrhosis) from the CATCH-LIFE cohort in China were included. The relationships between different AST/ALT ratios and the risk of adverse 90-day outcomes (death or liver transplantation) were determined in patients with cirrhosis or hepatitis B virus (HBV)-associated advanced fibrosis, respectively.

**Results:**

In the patients with HBV-associated advanced fibrosis, the risk of 90-day adverse outcomes increased with AST/ALT ratio; after adjusting for all confounding factors, the risk of adverse 90-day outcomes was the highest when AST/ALT ratio was more than 1.08 (OR = 6.91 [95% CI = 1.789–26.721], *p* = 0.005), and the AST/ALT ratio of >1.9 accelerated the development of adverse outcomes. In patients with cirrhosis, an AST/ALT ratio > 1.38 increased the risk of adverse 90-day outcomes in all univariables (OR = 1.551 [95% CI = 1.216–1.983], *p* < 0.001) and multivariable-adjusted analyses (OR = 1.847 [95% CI = 1.361–2.514], *p* < 0.001), and an elevated AST/ALT ratio (<2.65) accelerated the incidence of 90-day adverse outcomes. An AST/ALT ratio of >1.38 corresponded with a more than 20% incidence of adverse outcomes in patients with cirrhosis.

**Conclusion:**

The AST/ALT ratio is an independent risk factor for adverse 90-day outcomes in patients with cirrhosis and HBV-associated advanced fibrosis. The cutoff values of the AST/ALT ratio could help clinicians monitor the condition of patients when making clinical decisions.

## Introduction

Chronic liver disease (CLD) is an extremely common clinical condition primarily caused by hepatitis virus infection, alcohol abuse, toxic injuries, autoimmune diseases, genetic defects, and metabolic diseases (1). Some patients with CLD eventually develop liver fibrosis, cirrhosis, or liver malignancy, which are prominent threats to human health. Cirrhosis and liver fibrosis caused by CLD impose a substantial economic burden on many countries and have increased worldwide over the past 30 years (2). The major type of CLD in the Asia-Pacific region is chronic viral hepatitis, followed by alcoholic liver disease and non-alcoholic steatohepatitis (3); the latter two conditions are the main types of CLD in Western countries (4).

Acute exacerbations of CLD often develop from one or more clearly defined acute hepatic insults, advanced liver fibrosis, or acute decompensation (AD) of cirrhosis (liver cirrhosis with acute complications such as acute development of ascites, hepatic encephalopathy, gastrointestinal hemorrhage, infection, or any combination thereof, requiring hospitalization) (4). A high proportion of patients with cirrhosis or advanced liver fibrosis frequently develop liver and/or extrahepatic organ failure(s) with high short-term mortality rates. A recent study reported that the burden of liver fibrosis is associated with an unfavorable long-term prognosis, and advanced liver fibrosis is associated with an increased risk of all-cause mortality, with a cumulative all-cause mortality rate of 6.4% (5). In patients with cirrhosis, acute insults manifest as cirrhosis and often result in complications (e.g., ascites, gastrointestinal bleeding, HE, and/or bacterial infections) (6). Acute exacerbation of CLD can be a serious threat to the lives of patients. The short-term mortality rate in patients with AD ranges from 40 to 80% (4, 7). However, only a limited number of studies have investigated the relationship between advanced liver fibrosis or cirrhosis and the short-term prognosis in patients with severe liver damage.

Aspartate aminotransferase (AST) is expressed in various tissues, whereas alanine aminotransferase (ALT) is predominantly expressed in the liver. Serums AST and ALT are routine laboratory markers of liver injury and these two parameters are important indices for assessing the degree of hepatocellular injury in clinical practice (8). De Ritis et al. first reported the ratio of serum AST to ALT levels in 1957 (8). This ratio represents the degrees of liver injury. When the ratio of AST/ALT is less than 1, it indicates the liver injury is considered mild. In contrast, when the ratio is more than 1, the degree of hepatocyte damage is considered severe. In recent years, this ratio has been widely used to evaluate the prognosis of diseases unrelated to the liver (9–13). Previous studies have reported that the AST/ALT ratio can predict cirrhosis and hepatic fibrosis in patients with viral hepatitis (14, 15). Two recent studies involving patients with hepatocellular carcinoma demonstrated that an elevated AST/ALT ratio predicts poor outcomes (16, 17). However, the relationship between the AST/ALT ratio and short-term adverse outcomes (mortality or liver transplantation) in patients with liver fibrosis or cirrhosis with acute exacerbation remains unknown.

Advanced liver fibrosis and cirrhosis represent two different degrees of liver injury, and the AST/ALT ratio might present different levels, which may be significant to the prognosis of advanced liver fibrosis and cirrhosis. Currently no data regarding the relationship between the AST/ALT ratio and 90-day risk of adverse outcomes in patients with acute exacerbation of liver fibrosis or cirrhosis are available. Therefore, this study evaluated the association between AST/ALT ratio and risk of adverse 90-day outcomes (mortality or liver transplantation) in a prospective multicenter cohort of patients with advanced liver fibrosis and cirrhosis in China.

## Methods

### Patients

All data used in this study were obtained from the Chinese Acute on Chronic Liver Failure (CATCH-LIFE) Study, a prospective multicenter cohort study of patients with acute exacerbation of chronic liver disease from 14 hospitals throughout China. This study is registered at Shanghai (NCT02457637 and NCT03641872).^1^ The ethics committees of all participating hospitals approved the study, and all patients provided written informed consent. A total of 3,970 patients with CLD (cirrhosis or non-cirrhosis) hospitalized for acute exacerbation (AD and/or acute liver injury) were enrolled (2,600 patients hospitalized from January 2015 to December 2016 and 1,370 patients hospitalized from July 2018 to January 2019) (18, 19). All patients were followed-up for at least 90 days. In the CATCH-LIFE Study, patients with CLD (cirrhosis or non-cirrhosis) hospitalized for acute exacerbation were required to meet the following inclusion criteria: (1) acute liver injury, ALT or AST level of more than three times the upper limit of the normal level, or total bilirubin (TBIL) level of more than twice the upper limit of the normal level within 1 week before enrollment; and (2) AD of cirrhosis, including HE, gastrointestinal bleeding (variceal bleeding), jaundice (TBIL level of >5 mg/dL), ascites, and bacterial infection within 1 month before enrollment (19). The exclusion criteria were as follows: (1) severe chronic extrahepatic disease; (2) primary liver cancer or other liver malignancies before or during admission; (3) extrahepatic malignancies; and (4) age < 18 or > 80 years and pregnancy. All patients were monitored after diagnosis, and the outcomes (survival, mortality, or liver transplantation) of all patients within the 90-day follow-up period were evaluated. Adverse 90-day outcomes refer to death or the need for liver transplantation within 90 days.

Patients with cirrhosis or advanced fibrosis were enrolled and divided into two groups. Cirrhosis was diagnosed based on the history of liver disease, clinical symptoms, computed tomography/magnetic resonance imaging, abdominal ultrasound, and laboratory test results. Based on a history of CLD prior to the onset of the present episode, advanced fibrosis was defined as a fibrosis-4 (FIB-4) score of ≥1.45 but with no cirrhosis (20). Patients without cirrhosis with only mild or no fibrosis and an FIB-4 score of <1.45 were excluded from the study.

### Data collection

The following data were collected from all enrolled patients: clinical symptoms, related complications, demographic characteristics, baseline laboratory measurements, and imaging examination results. Demographic characteristics and baseline laboratory measurements included sex, age, survival time, AST(U/L) and ALT(U/L) levels, TBIL level (mg/dL), alkaline phosphatase level (U/L), glutamyl transpeptidase level (U/L), serum creatinine level (mg/dL), white blood cell count, neutrophil count, lymphocyte count, hemoglobin level, platelet count, and international normalized ratio (INR). Related complications included hyponatremia, HE, and hepatorenal syndrome. The model for end-stage liver disease (MELD) score was calculated from the laboratory data (21). The following equations were used to determine the AST/ALT ratio, MELD score and FIB-4:

- AST/ALT ratio = AST (IU/l)/ALT (IU/l).
- MELD score = 3.78 × ln serum bilirubin level (mg/dL) + 11.2 × ln (INR) + 9.57 × ln serum creatinine (mg/dL) + 6.43.
- FIB-4 = age (years) × AST (IU/l)/[PLT (10^9^/l) × ALT(IU/l)^1/2^].

### Statistical analysis

The baseline characteristics of the patients were compared across quartiles of the AST/ALT ratio. Continuous variables were presented as means ± standard deviations or medians (minimum, maximum, and interquartile ranges), and categorical variables were presented as counts and percentages. The χ^2^ test was performed for categorical variables, and the one-way analysis of variance and Kruskal–Wallis test were performed for normally and non-normally distributed continuous variables, respectively.

According to the AST/ALT ratio quartiles, the cumulative survival rates were calculated using the Kaplan–Meier method and compared using the log-rank test. The AST/ALT ratio was analyzed with respect to the risk of adverse outcomes across quartiles (with the incidence of adverse outcomes in the lowest quartile cohort as the reference) and as a continuous variable. A logistic regression model was used to investigate the association between the AST/ALT ratio and 90-day prognosis. Important risk factors for the prognosis of chronic liver disease were selected to adjust the AST/ALT ratio. First, the AST/ALT ratio in the quartiles or as a continuous variable was not adjusted for the variables. Second, all potential risk factors were added to the model, including age, sex, HE grade, infection, ascites, gastrointestinal bleeding, total bilirubin, INR, and creatinine.

Subgroup analyses stratified by age, sex, etiology, INR, and TBIL levels were performed using forest plots. The adverse 90-day outcomes corresponding to the AST/ALT ratio were plotted as a “correlation curve” using the generalized additive model (GAM). The second derivative (acceleration) of the AST/ALT ratio was used to describe the non-linear relationship between the AST/ALT ratio and adverse 90-day outcomes. All analyses were conducted using SPSS (version 22.0) for Windows (Chicago) and R (version 3.6.0), and all hypothesis tests were performed using a two-tailed analysis. A *p* value of <0.05 was considered statistically significant.

## Results

### Study participants

**Figure 1:**
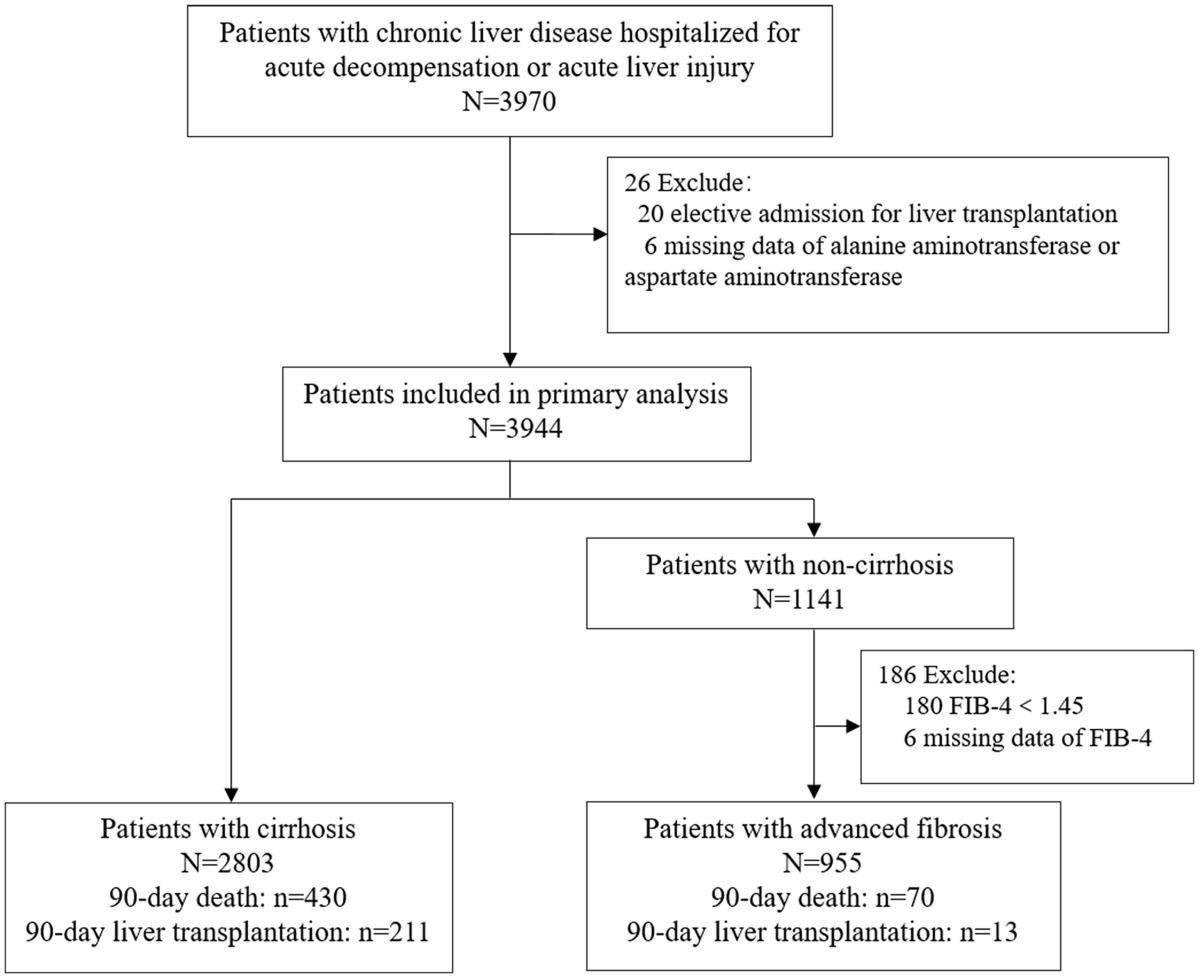
Flowchart of enrollment of patients with cirrhosis and advanced fibrosis.

A total of 3,758 patients were included in this study (Figure 1). of which 955 (25.4%) had advanced fibrosis and 2,803 (74.6%) had cirrhosis. Patients who underwent liver transplantation within the past 48 h (*n* = 20), missing ALT or AST values (*n* = 6) and FIB-4 scores (*n* = 16), or without cirrhosis and FIB-4 scores <1.45 (*n* = 180) were excluded. The missing data are shown in Supplementary Figure S1. All the participants were followed up for at least 90 days. The cumulative adverse 90-day outcome rate of the patients with advanced fibrosis was 8.7% (83/955), mortality rate was 7.3% (70/955), and transplantation rate was 1.4% (13/955; Table 1; Figure 1). The cumulative adverse 90-day outcome rate in patients with cirrhosis was 22.9% (641/2803), mortality rate was 15.3% (430/2803), and transplantation rate was 7.5% (211/2803; Table 2; Figure 1). The baseline and demographic characteristics of the patients with advanced fibrosis and cirrhosis are presented in Tables 1, 2.

**Table 1:** Comparison of the baseline characteristics of the patients with advanced fibrosis according to the aspartate aminotransferase/alanine aminotransferase ratio quartiles at admission.

Characteristics	Groups by ASL/ALT ratio quartiles (*N* = 955)				*p* value
	Q1:≤0.50	Q2:(0.50, 0.69]	Q3:(0.69, 1.08]	Q4:>1.08	
	*N* = 246	*N* = 238	*N* = 232	*N* = 239	
Demographics					
Age, mean (SD)	40.8 (10.7)	40.3 (11.2)	45.4 (12.2)	46.8 (12.7)	<0.001
Gender, No. (%)	213 (86.6)	187 (78.6)	167 (72.0)	153 (64.0)	<0.001
Etiology, No. (%)					<0.001
HBV	192 (78.0)	187 (78.6)	170 (73.3)	150 (62.8)	
Alcoholic	2 (0.8)	3 (1.3)	2 (0.9)	19 (7.9)	
Others	52 (21.1)	48 (20.2)	60 (25.9)	70 (29.3)	
Complications, No. (%)					
Ascites	17 (6.9)	23 (9.7)	36 (15.5)	50 (20.9)	<0.001
Bacterial Infection	18 (7.3)	15 (6.3)	35 (15.1)	40 (16.7)	<0.001
Hepatic encephalopathy					0.490
Not overt	238 (96.7)	230 (96.6)	223 (96.1)	225 (94.1)	
Grade1	2 (0.8)	4 (1.7)	5 (2.2)	6 (2.5)	
Grade2	3 (1.2)	0 (0.0)	2 (0.9)	6 (2.5)	
Grade3	2 (0.8)	2 (0.8)	1 (0.4)	2 (0.8)	
Grade4	1 (0.4)	2 (0.8)	1 (0.4)	0 (0.0)	
Laboratory results, median (IQR)					
Total bilirubin, mg/dL	4.8 (1.7,13.3)	3.8 (1.7,12.5)	8.6 (2.5,16.7)	8.6 (3.1,18.2)	<0.001
International normalized ratio	1.2 (1.0,1.5)	1.2 (1.1,1.6)	1.3 (1.1,1.7)	1.4 (1.1,1.8)	<0.001
Creatinine, mg/dL	0.8 (0.7,0.9)	0.8 (0.7,0.9)	0.7 (0.6,0.9)	0.8 (0.6,0.9)	0.131
Blood urea nitrogen, mmol/dL	3.8 (3.1,4.6)	3.8 (3.1,4.6)	4.0 (3.1,4.9)	3.9 (3.1,5.2)	0.379
Albumin, g/L	37.4 (33.6,41.3)	38.0 (34.2,41.4)	35.3 (31.4,39.4)	33.8 (30.0,38.3)	<0.001
White blood cell, 10^9^/L	5.0 (4.1,6.7)	5.2 (4.3,6.3)	5.7 (4.4,7.3)	5.4 (4.2,7.4)	0.018
Platelet, 10^9^/L	138.5 (116.0,168.0)	139.0 (106.2,175.0)	128.0 (92.0,181.1)	132.0 (92.5,179.0)	0.189
Hemoglobin, g/L	141.0 (133.0,153.8)	143.5 (130.0,153.0)	132.0 (120.5,145.1)	121.0 (107.0,136.5)	<0.001
Sodium, mmol/L	139.3 (137.6,141.1)	139.8 (137.0,141.0)	138.8 (136.0,141.1)	138.0 (135.5,140.8)	<0.001
Alanine aminotransferase, U/L	781.5 (500.8, 1180.9)	684.0 (329.2, 1253.8)	584.5 (207.4, 1036.2)	149.0 (65.0, 449.5)	<0.001
Aspartate aminotransferase, U/L	293.2 (179.4, 441.0)	431.4 (198.5, 762.8)	463.0 (183.0, 888.0)	239.0 (116.4, 695.3)	<0.001
Score, mean (SD)					
MELD	15.5 (7.9)	15.5 (7.2)	17.8 (7.3)	18.2 (8.0)	<0.001
MELD-Na	15.8 (8.3)	16.0 (7.7)	18.5 (7.8)	19.1 (8.7)	<0.001
CTP	7.1 (1.9)	7.1 (1.9)	7.8 (2.0)	8.2 (2.0)	<0.001
90-day Outcome, No. (%)					<0.001
Survival	235 (95.5)	226 (95.0)	209 (90.1)	202 (84.5)	
Death	10 (4.1)	11 (4.6)	20 (8.6)	29 (12.1)	
Transplantation	1 (0.4)	1 (0.4)	3 (1.3)	8 (3.3)	

SD, standard deviation; HBV, hepatitis B virus; IQR, interquartile range; MELD, the model of end-stage liver disease; MELD-Na, the model of end-stage liver disease with sodium; CTP, Child-Turcotte-Pugh.

**Table 2:** Comparison of the baseline characteristics of the patients with cirrhosis according to the aspartate aminotransferase/alanine aminotransferase ratio quartiles at admission.

Characteristics	Groups by ASL/ALT ratio quartiles (*N* = 2,803)				*p* value
	Q1:≤0.91	Q2:(0.91, 1.33]	Q3:(1.33, 1.85]	Q4:>1.85	
	*N* = 702	*N* = 706	*N =* 698	*N* = 697	
Demographics					
Age, mean (SD)	48.2 (11.4)	51.3 (10.9)	53.0 (11.5)	52.9 (11.3)	<0.001
Gender, No. (%)	585 (83.3)	515 (72.9)	470 (67.3)	474 (68.0)	<0.001
Etiology, No. (%)					<0.001
HBV	519 (73.9)	485 (68.7)	423 (60.6)	338 (48.5)	
Alcoholic	47 (6.7)	47 (6.7)	69 (9.9)	146 (20.9)	
Others	136 (19.4)	174 (24.6)	206 (29.5)	213 (30.6)	
Complications, No. (%)					
Ascites	371 (52.8)	413 (58.5)	454 (65.0)	477 (68.4)	<0.001
Gastrointestinal bleeding	114 (16.2)	166 (23.5)	164 (23.5)	132 (18.9)	0.001
Bacterial Infection	166 (23.6)	173 (24.5)	169 (24.2)	215 (30.8)	0.006
Hepatic encephalopathy					0.065
Not overt	633 (90.2)	647 (91.6)	625 (89.5)	601 (86.2)	
Grade1	25 (3.6)	20 (2.8)	29 (4.2)	36 (5.2)	
Grade2	28 (4.0)	26 (3.7)	35 (5.0)	35 (5.0)	
Grade3	11 (1.6)	8 (1.1)	8 (1.1)	20 (2.9)	
Grade4	5 (0.7)	5 (0.7)	1 (0.1)	5 (0.7)	
Laboratory results, median (IQR)					
Alanine aminotransferase, IU/L	217.0 (69.0, 558.8)	60.4 (31.0, 154.8)	39.8 (22.8, 78.6)	27.4 (16.0, 53.0)	<0.001
Aspartate aminotransferase, IU/L	117.2 (42.6, 289.0)	69.5 (35.6, 172.5)	61.0 (35.0, 119.2)	73.0 (43.0, 143.1)	<0.001
Total bilirubin, mg/dL	8.7 (1.9,19.7)	2.9 (1.3,12.2)	2.9 (1.5,9.3)	4.5 (1.8,12.4)	<0.001
International normalized ratio	1.5 (1.2,2.1)	1.4 (1.2,1.8)	1.5 (1.3,1.8)	1.5 (1.3,1.9)	<0.001
Creatinine, mg/dL	0.8 (0.6,0.9)	0.8 (0.7,0.9)	0.8 (0.6,1.0)	0.8 (0.6,1.0)	0.018
Blood urea nitrogen, mmol/dL	4.6 (3.5,6.2)	5.3 (3.9,7.3)	5.0 (3.8,7.7)	5.3 (3.7,8.1)	<0.001
Albumin, g/L	32.1 (29.2,36.0)	31.0 (27.2,34.6)	30.1 (26.0,33.8)	28.7 (25.3,32.5)	<0.001
White blood cell, 10^9^/L	5.2 (3.8,7.1)	4.4 (3.0,6.8)	4.4 (3.0,6.6)	5.0 (3.3,7.4)	<0.001
Platelet, 10^9^/L	91.0 (58.0,133.0)	68.0 (46.5,103.0)	70.0 (46.0,105.0)	74.5 (49.0,112.2)	<0.001
Hemoglobin, g/L	125.0 (107.0,138.0)	111.0 (89.0,127.0)	104.0 (83.8,118.0)	97.0 (82.0,113.2)	<0.001
Sodium, mmol/L	138.3 (135.3,141.0)	138.1 (135.1,141.0)	138.0 (134.3,141.0)	136.9 (133.0,139.3)	<0.001
Score, mean (SD)					
MELD	18.9 (8.6)	16.4 (8.4)	16.5 (8.0)	17.7 (8.2)	<0.001
MELD-Na	19.9 (9.0)	17.5 (9.2)	17.6 (8.7)	19.7 (8.5)	<0.001
CTP	9.1 (2.2)	8.9 (2.1)	9.2 (2.0)	9.7 (1.9)	<0.001
90-day Outcome, No. (%)					<0.001
Survival	555 (79.1)	568 (80.5)	545 (78.1)	494 (70.9)	
Death	113 (16.1)	89 (12.6)	92 (13.2)	136 (19.5)	
Transplantation	34 (4.8)	49 (6.9)	61 (8.7)	67 (9.6)	

SD, standard deviation; HBV, hepatitis B virus; IQR, interquartile range; MELD, the model of end-stage liver disease; MELD-Na, the model of end-stage liver disease with sodium; CTP, Child-Turcotte-Pugh.

### AST/ALT ratio associated with adverse 90-day outcomes

**Figure 2:**
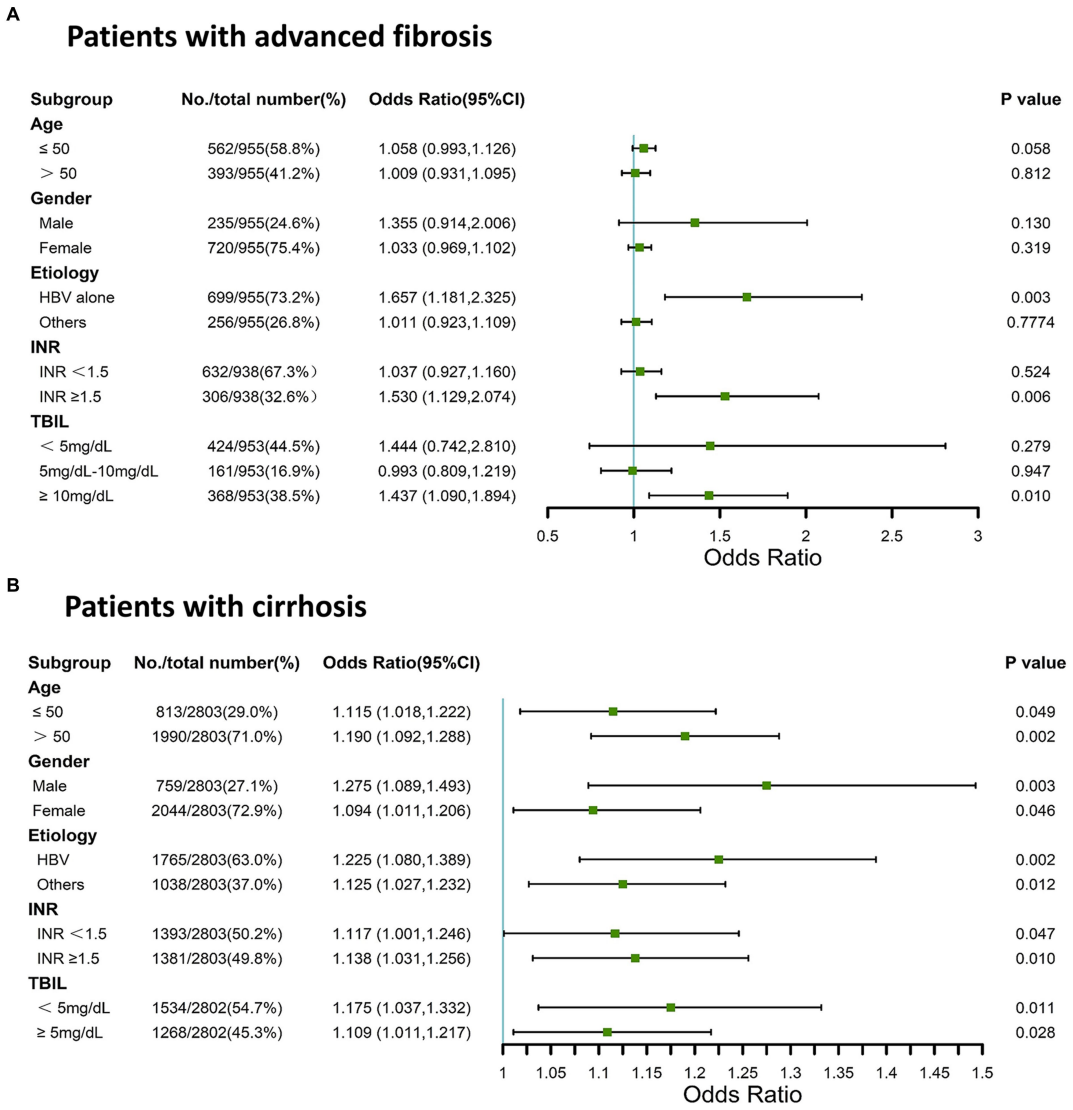
Stratified analysis using forest plots for AST/ALT ratio and adverse 90-day outcomes in patients with cirrhosis and advanced fibrosis. **(A)** Patients with advanced fibrosis; **(B)** Patients with cirrhosis. AST, aspartate aminotransferase; ALT, alanine aminotransferase; INR, international normalized ratio; TBIL, total bilirubin; HBV, Hepatitis B virus.

The association between the AST/ALT ratio and adverse 90-day outcomes may be confounded by age, sex, pathological basis, and other factors. Thus, we analyzed the association between AST/ALT ratio and adverse outcomes by stratifying patients according to multiple clinical parameters. In the stratified analyses, we found that the AST/ALT ratio was associated with adverse 90-day outcomes in the patients with advanced fibrosis with an INR of ≥1.5, or a TBIL level of >10 mg/dL, or an HBV infection {Figure 2A; INR of ≥1.5: odds ratio [OR] = 1.530 (95% CI = 1.129–2.074), *p* = 0.006; TBIL level of ≥10 mg/dL: OR = 1.437 (95% CI = 1.090–1.894), *p* = 0.010; HBV infection: OR = 1.657 (95% CI = 1.181–2.325), *p* = 0.003}. Figure 2B shows that the AST/ALT ratio was associated with the risk of mortality or liver transplantation within 90 days in patients with cirrhosis, irrespective of age, sex, etiology, TBIL level, or INR (Figure 2B; all *p* < 0.05). Thus, the AST/ALT ratio was associated with adverse 90-day outcomes in patients with cirrhosis and HBV-associated advanced fibrosis.

### AST/ALT ratio as an independent risk factor for adverse 90-day outcomes

To further explore the relationship between the AST/ALT ratio and adverse 90-day outcomes in patients with HBV-associated advanced fibrosis and cirrhosis, we used logistic regression to analyze the influence of the AST/ALT ratio on the prognosis of patients after multiple interpolations, including the AST/ALT ratio alone and adjusted AST/ALT ratio in the multivariable analysis.

**Figure 3:**
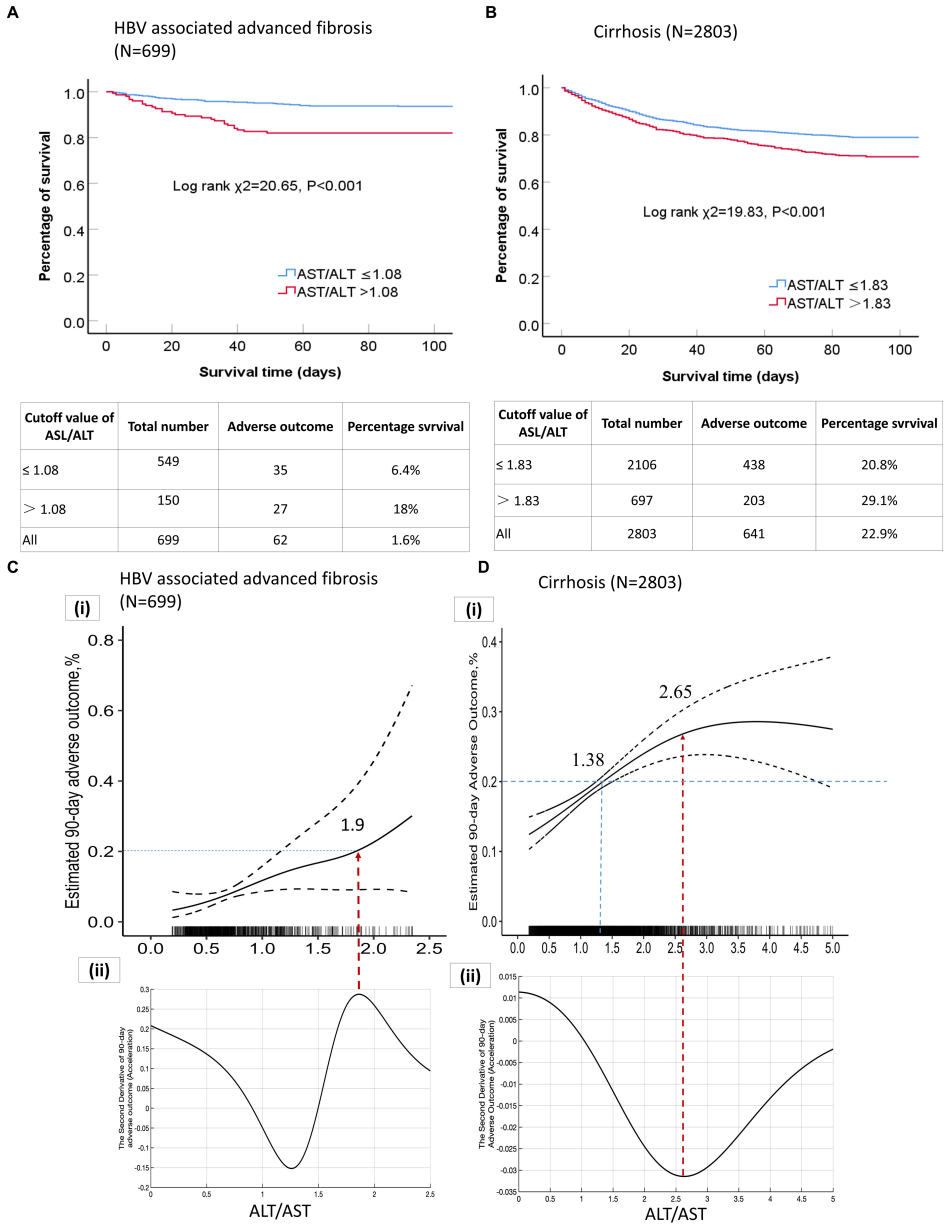
**(A)** Kaplan–Meier curves of patients with HBV-associated advanced fibrosis based on the AST/ALT ratio cut-off value; **(B)** Kaplan–Meier curves of patients with cirrhosis based on the AST/ALT ratio cutoff value; **(C) (a)** Correlation curve for the AST/ALT ratio and adverse 90-day outcomes in patients with HBV-associated advanced fibrosis; **(C)** **(b)** Second derivative (acceleration) of the AST/ALT ratio relative to adverse 90-day outcomes; **(D) (a)** Correlation curve for the AST/ALT ratio and adverse 90-day outcomes in patients with cirrhosis; **(D)** **(b)** Second derivative (acceleration) of the AST/ALT ratio relative to adverse 90-day outcomes. AST, aspartate aminotransferase; ALT, alanine aminotransferase; HBV, Hepatitis B virus.

In patients with HBV-associated advanced fibrosis, the AST/ALT ratio, as a continuous variable, influenced adverse 90-day outcomes (unadjusted OR = 1.65 [95% CI = 1.181–2.325], *p* = 0.003; adjusted OR = 1.42 [95% CI = 1.051–1.908], *p* = 0.022; Table 3). The categorical data analysis showed that the risk of adverse 90-day outcomes significantly increased with an AST/ALT ratio of >0.5 compared with an AST/ALT ratio of ≤0.5. After adjusting for all confounding factors that may affect the outcomes, the risk of adverse 90-day outcomes was the highest when the AST/ALT ratio was >1.08. Compared to patients whose AST/ALT ratio was ≤0.50, the models showed that the ORs for adverse 90-day outcomes in those with an AST/ALT ratio > 1.08 were 4.46-fold at non-adjustment (95% CI = 2.029–9.817, *p* < 0.001; Table 3) and 6.91-fold after multivariable adjustment (95% CI = 1.789–26.721, *p* = 0.005; Table 3). The 90-day mortality of patients with HBV-advanced fibrosis (AST/ALT ratio > 1.08) was significantly higher than that in patients with AST/ALT ratio ≤ 1.08 (χ^2^ = 20.65, *p* < 0.001; Figure 3A).

**Table 3:** Unadjusted and adjusted odds ratios of the adverse 90-day outcomes relative to the aspartate aminotransferase/alanine aminotransferase ratio in the patients with HBV-associated advanced fibrosis.

Aspartate aminotransferase/Alanine aminotransferase ratio	Number of patients	Number of adverse outcome (%)	Odds ratios of adverse outcome (95%CI), *p* value	
			Unadjusted	Adjusted*
As a continuous variable	699	62(8.9)	1.65 (1.181–2.325), 0.003	1.42 (1.051–1.908), 0.022
As a categorical variable				
≤0.50	192	9 (4.7)	1 (Reference)	1 (Reference)
(0.50,0.69]	187	8 (4.3)	1. 91 (0.343–2.408), 0.847	2.21 (0.478–10.172), 0.311
(0.69,1.08]	170	18 (10.6)	2.41 (1.051–5.514), 0.038	3.29 (0.793–13.664), 0.100
>1.08	150	27 (18.0)	4.46 (2.029–9.817), < 0.001	6.91 (1.789–26.721), 0.005

*Adjusted for age, gender, hepatic encephalopathy grade, infection, ascites, gastrointestinal bleeding, total bilirubin, international normalized ratio, creatinine and alanine transaminase; 95%CI, 95% confidence interval.

In patients with cirrhosis, an elevated AST/ALT ratio was independently associated with an increased risk of adverse outcomes. The AST/ALT ratio, as a continuous variable, was a risk factor for adverse 90-day outcomes in the univariable- (OR = 1.13[95% CI = 1.055–1.222], *p* = 0.001; Table 4) and multivariable-adjusted analyses (OR = 1.11 [95% CI = 1.021–1.217], *p* = 0.014; Table 4). The categorical data analysis showed that the risk of adverse 90-day outcomes increased with an AST/ALT ratio of >1.33 compared with an AST/ALT ratio of ≤0.91. An AST/ALT ratio of >1.83 was a risk factor for adverse 90-day outcomes in all univariable- (OR = 1.55 [95% CI = 1.216–1.983], *p* < 0.001; Table 4) and multivariable-adjusted analyses (OR = 1.85 [95% CI = 1.361–2.514], *p* < 0.001; Table 4). The 90-day mortality of patients with cirrhosis (AST/ALT ratio > 1.83) was significantly higher than that in patients with AST/ALT ratio ≤ 1.83 (χ^2^ = 19.83, *p* < 0.001; Figure 3B).

**Table 4:** Unadjusted and adjusted odds ratios of the adverse 90-day outcomes relative to the aspartate aminotransferase/alanine aminotransferase ratio in the patients with cirrhosis.

Aspartate aminotransferase/Alanine aminotransferase ratio	Number of patients	Number of adverse outcome(%)	Odds ratios of adverse outcome (95%CI), *p* value	
			Unadjusted	Adjusted*
As a continuous variable	2,803	641 (22.9)	1.13 (1.055–1.222), 0.001	1.11 (1.021–1.217), 0.014
As a categorical variable				
≤0.91	702	147 (20.9)	1 (Reference)	1 (Reference)
(0.91,1.33]	706	138 (19.5)	0.92 (0.707–1.190), 0.515	1.25 (0.913–1.700), 0.167
(1.33,1.83]	698	153 (21.9)	1.06 (0.821–1.369), 0.655	1.42(1.044–1.945), 0.026
>1.83	697	203 (29.1)	1.55 (1.216–1.983), <0.001	1.85 (1.361–2.514), <0.001

*Adjusted for age, gender, etiology, hepatic encephalopathy grade, infection, ascites, gastrointestinal bleeding, total bilirubin, international normalized ratio, creatinine, serum sodium and alanine transaminase; 95%CI, 95% confidence interval.

### AST/Alt ratio of >1.9 accelerated the adverse 90-day outcomes in patients with HBV-associated advanced fibrosis

The GAM and spline illustrated the relationship between AST/ALT ratios and multivariable-adjusted adverse 90-day outcomes in patients with HBV-associated advanced fibrosis. The correlation curves for the AST/ALT ratio and adverse 90-day outcomes in patients with HBV-associated advanced fibrosis increased monotonically; however, the rate of change in the adverse outcomes per AST/ALT ratio was not constant (Figures 3C,a). We determined the AST/ALT ratio corresponding to an adverse outcome rate of 20%. The AST/ALT ratio of 1.9 corresponded to a 20% incidence of adverse outcomes. The correlation curve for the AST/ALT ratio and adverse outcomes in patients with HBV-associated advanced fibrosis was divided into two parts by an inflection point (AST/ALT ratio = 1.9, the peak in Figures 3C,b of the second derivative). The peak points in the second derivative corresponded to the maximum values of the acceleration of adverse outcomes relative to the change in the AST/ALT ratio. An AST/ALT ratio > 1.9 accelerated the adverse 90-day outcomes, and absolute incidence of adverse outcomes resulting from a 0.1 increase in the AST/ALT ratio was the largest (Figures 3C,a,b).

We plotted the correlation curves for the AST/ALT ratios and adverse outcomes in patients with HBV-associated advanced fibrosis with an INR of ≥1.5 and/or a TBIL level of ≥10 mg/dL. The trends observed in the three curves were similar and nonlinear, and all three curves had similar inflection points. The AST/ALT ratio ≥ 1.8 or 1.9 accelerated the risk of 90-day adverse outcomes in patients with HBV-associated advanced fibrosis with severe jaundice and/or coagulopathy (Supplementary Figures S2A–C).

### An elevated AST/Alt ratio (<2.65) accelerated the incidence of adverse 90-day outcomes inpatients with cirrhosis

GAM and spline illustrated the relationship between the AST/ALT ratio and multivariable-adjusted adverse 90-day outcomes in patients with cirrhosis. The correlation curve for the AST/ALT ratio and adverse 90-day outcomes in patients with cirrhosis monotonically increased with AST/ALT ratios <2.65, and the rate of change in the adverse 90-day outcomes per AST/ALT ratio was not constant, implying a nonlinear correlation (Figures 3D,a). When the AST/ALT ratio was <2.65, the incidence of adverse 90-day outcomes in patients with cirrhosis increased with an increase in the AST/ALT ratio; however, the acceleration of the increase was gradually attenuated (Figures 3D,b). An increase in the AST/ALT ratio per 0.1 units resulted in a smaller increase in adverse outcomes. An AST/ALT ratio of 2.65 corresponded to the minimum value of acceleration in the second derivative (Figures 3D,b), and incidence rate of adverse outcomes reached a maximum. When the AST/ALT ratio was >2.65, the incidence of adverse outcomes stopped increasing, indicated a saturation effect.

### The AST/Alt ratio of >1.38 corresponded with a more than 20% incidence of adverse outcomes in the patients with cirrhosis

We attempted to determine the AST/ALT ratio corresponding to an adverse 90-day outcome rate of 20% in patients with cirrhosis. When the incidence of adverse outcomes in patients with cirrhosis was 20%, the corresponding cut-off AST/ALT ratio was 1.38 (Figures 3D,a). We compared the characteristics of the patients with cirrhosis who had an AST/ALT ratio of ≤1.38 and > 1.38 (Supplementary Table S1). The patients with an AST/ALT ratio of ≤1.38 were predominantly male, were of age < 50 years and had more severe liver injury than the patients with an AST/ALT ratio of >1.38. The mean AST, ALT, and TBIL levels of the patients with an AST/ALT ratio of ≤1.38 were significantly higher than those of the patients with an AST/ALT ratio of >1.38. Furthermore, the 28-day and 90-day mortality and liver transplantation rates of the patients with an AST/ALT ratio of ≤1.38 were lower than those of the patients with an AST/ALT ratio of >1.38 (Supplementary Figure S3; Supplementary Table S1; all *p* < 0.05). Patients with an AST/ALT ratio > 1.38 tended to be older (>50 years) and had a higher incidence of decompensated cirrhosis-associated complications, such as ascites, infection, hyponatremia, HE, and high creatinine levels. Therefore, mortality and liver transplantation rates were higher in cirrhotic patients with AST/ALT ratio more than 1.38 than those patients with an AST/ALT ratio ≤ 1.38.

## Discussion

This large prospective cohort study is the first to investigate the correlation between AST/ALT ratio and the risk of adverse 90-day outcomes in patients with cirrhosis and advanced fibrosis. The main finding of our study was that increased AST/ALT ratio increased the risk of adverse outcomes in patients with cirrhosis and HBV-associated advanced fibrosis. Based on the large sample size and sufficient data from the cohort, we calculated different clinical AST/ALT cut-off ratios for cirrhosis and HBV-associated advanced fibrosis. We found that an AST/ALT ratio of >1.9 accelerated the incidence of adverse outcomes in the patients with HBV-associated advanced fibrosis or combined with coagulopathy (INR ≥1.5); whereas an AST/ALT ratio of >1.8 increased the risk of adverse outcomes in the patients with HBV-associated advanced fibrosis combined with severe jaundice. Moreover, the risk of adverse outcomes increased with an AST/ALT ratio of <2.65, and incidence of adverse 28-day and 90-day outcomes significantly increased with an AST/ALT ratio ranged from 1.38 to 2.65 in patients with cirrhosis. Thus, AST/ALT ratio was independently associated with adverse 90-day outcomes in patients with cirrhosis and HBV-associated advanced fibrosis.

The serum transaminase levels and AST/ALT ratios have been predominantly used as markers for assessing the severity of liver disease and as predictive factors for cirrhosis (14). The relationship between AST/ALT ratio and the prognosis of CLD with acute exacerbation, especially AD of cirrhosis and advanced fibrosis, has not been extensively evaluated. Very few studies, mostly with a sample size of <100, have reported that the AST/ALT ratio is associated with an increased risk of mortality in patients with autoimmune hepatitis and that it can predict the prognosis of liver cirrhosis of viral etiology (22). In addition, the AST/ALT ratio is included in the FIB-4 score, which is used for the noninvasive diagnosis of liver fibrosis (20); however, the effect of an elevated AST/ALT ratio on the risk of mortality or liver transplantation is unknown. The CATCH-LIFE study is a large prospective multicenter cohort study of patients with diverse non-cirrhosis or cirrhosis CLD. Therefore, this study provides an estimate of the relationship between AST/ALT ratio and adverse outcomes that are more comprehensive in cirrhosis and advanced fibrosis, which manifest as different causes and states in the general population.

The results of this study provide evidence of a close relationship between AST/ALT ratio and adverse outcomes in individuals with CLD.

In patients with HBV-associated advanced fibrosis, the significant association between the AST/ALT ratio and risk of adverse 90-day outcomes was not altered, even after adjustment in the multivariable logistic regression models. Hepatocellular damage or death results in the release of AST and ALT from the hepatocytes (23). ALT is predominantly present in the cytoplasm of hepatocytes, whereas AST is present in the hepatocyte cytoplasm and mitochondria (24). AST and ALT are biomarkers that reflect liver injury in liver diseases. The levels of AST and ALT in the serum are similar, leading to an AST/ALT ratio of approximately 1 in healthy individuals (25). Notably, an increased AST/ALT ratio reflects progressive liver damage. A previous study reported that AST/ALT and MELD scores were significantly higher in patients who died during 3-month and 1-year follow-ups than in those who survived (18). In our study, we found that an AST/ALT ratio > 1.9 accelerated the development of adverse outcomes in patients with HBV-associated advanced fibrosis, especially those with severe jaundice and/or coagulopathy, and that an elevated AST/ALT ratio was a high-risk factor for such outcomes. We also found that the risk of adverse outcomes increased with an AST/ALT ratio of <2.65 in the patients with cirrhosis, and the incidence of adverse 90-day outcome increased to >20% with an AST/ALT ratio of >1.38.

The potential mechanisms underlying the relationship between increased AST/ALT ratio and adverse outcomes are not entirely understood. Generally, as a marker of the severity of liver injury, the AST/ALT ratio may indicate a higher likelihood of mortality in patients with more complicated illnesses. In our study, the incidences of HE, infection, ascites, and gastrointestinal bleeding increased relative to the AST/ALT ratio, from the lowest to the highest values, and the MELD score also increased with an increase in the AST/ALT ratio. The AST/ALT ratio was positively associated with the risk of mortality and liver transplantation, even after adjusting for many clinical parameters and risk factors for mortality, implying a specific role of the AST/ALT ratio in the poor prognosis of cirrhosis and HBV-associated advanced fibrosis. The AST/ALT ratio may indicate mitochondrial dysfunction and increased oxidative stress. Thus, the serum AST/ALT ratio should not only be considered a simple biomarker of liver damage but also a key indicator of the deterioration of systemic conditions, which are risk factors for mortality.

Another potential mechanism is that the AST/ALT ratio reflects abnormal glucose metabolism in the liver. Studies have shown that severe liver injury and liver failure lead to significantly abnormal glucose metabolism, and that there are two main metabolic pathways for abnormal glucose metabolism (18, 26). First, extra-mitochondrial glucose metabolism is abnormal, including the glycolysis and pentose phosphate pathways. In acute-on-chronic liver failure and advanced liver injury, innate immune cells release inflammatory factors to accelerate glycogenolysis, glycolysis, and the production of lactate and pyruvate, and then produce ribose and amino acids through the pentose phosphate pathway to compensate for the material consumption of acute inflammation. Second, abnormal glucose metabolism in the mitochondria mainly manifests as a tricarboxylic acid cycling disorder. Viral infections, consumption of drugs and alcohol, and other pathogenic factors can lead to mitochondrial dysfunction and damage, decreased oxidative phosphorylation, and glycolytic failure. AST plays a vital role in aerobic glycolysis because of its ability to relocate nicotinamide adenine dinucleotide hydrogen into the mitochondria through malate–aspartate shuffling (27). Moreover, glutaminolysis and pyruvate production are catalyzed by ALT (28). In severe liver injury, a large number of hepatocyte necrosis, mitochondrial damage, AST and ALT release into the blood, and abnormal glucose metabolism accelerate liver failure. Thus, the AST/ALT ratio may reflect the state of the liver metabolism.

Stratified analyses revealed that the risk of adverse 90-day outcomes relative to the AST/ALT ratio was significant in patients with acute exacerbations of CLD and liver cirrhosis. We found that the AST/ALT ratio was independent of adjustment for age, sex, and etiology, suggesting that other mechanisms may play an important role in the effect of the AST/ALT ratio on the short-term prognosis of these patients. In a previous study on patients with virus-related cirrhosis, the AST/ALT ratio was reported to have a prognostic capability that was not significantly different from that of the MELD score, and AST/ALT ratio in combination with the MELD score yielded increased medium-term prognostic accuracy (29). In this study, we found a significant association between the AST/ALT ratio and the risk of adverse 90-day outcomes in patients with cirrhosis, and this relationship was not affected by INR or TBIL levels in patients with cirrhosis. We observed that when the AST/ALT ratio was <2.65, the incidence of 90-day adverse outcomes in patients with cirrhosis increased with an increase in the AST/ALT ratio; however, the increase in acceleration was gradually attenuated. The incidence of adverse outcomes reached the maximum value when the AST/ALT ratio was 2.65. When the AST/ALT ratio was >2.65, the incidence of adverse outcomes stopped increasing, indicating a saturation effect. This finding could help clinicians identify patients with severe liver disease who are at a higher risk of developing short-term adverse outcomes and may require close monitoring.

Our study had a few limitations. First, as more than 70% of the patients enrolled in this study were chronically infected with HBV, the findings were limited to patients with non-HBV-associated advanced fibrosis. Thus, the relationship between the AST/ALT ratio and adverse 90-day outcomes in patients with non-HBV-associated advanced fibrosis remains unclear. Second, we analyzed the AST/ALT ratio obtained only at baseline and did not assess the changes in aminotransferase levels over time. We could not explore the relationship between dynamic changes in the AST/ALT ratio and 90-day outcomes of the patients in this study. Third, the associations between the AST/ALT ratio and 90-day outcomes were based on observational data, and causation could not be determined. Nevertheless, our study is the largest cohort study to report the prospective association between AST/ALT ratio and adverse outcomes in individuals with acute exacerbation of CLD. The large number of participants, prospective design, and relatively long follow-up duration adjusted for potential confounders and multiple important risk factors.

In conclusion, the results of this study provide evidence for a close relationship between AST/ALT ratios and 90-day adverse outcomes in individuals with CLD. This study suggests that the AST/ALT ratio is a risk factor for adverse 90-day outcomes (high mortality or liver transplantation) in patients with cirrhosis and HBV-associated advanced fibrosis. The cut-off values of the AST/ALT ratio provide useful information for clinicians to monitor the condition of patients when making clinical decisions.

## Data availability statement

The original contributions presented in the study are included in the article/Supplementary material, further inquiries can be directed to the corresponding authors.

## Author contributions

HuL: Formal analysis, Investigation, Methodology, Writing – original draft, Writing – review & editing. HaL: Conceptualization, Funding acquisition, Methodology, Project administration, Writing – review & editing. GD: Formal analysis, Project administration, Validation, Writing – review & editing. XZ: Conceptualization, Formal analysis, Supervision, Validation, Writing – review & editing. YH: Conceptualization, Formal analysis, Funding acquisition, Validation, Writing – review & editing. JC: Data curation, Investigation, Validation, Writing – review & editing. ZM: Data curation, Investigation, Writing – review & editing. YG: Data curation, Investigation, Project administration, Writing – review & editing. ZQ: Data curation, Project administration, Validation, Writing – review & editing. FL: Investigation, Project administration, Validation, Writing – review & editing. XL: Funding acquisition, Investigation, Project administration, Writing – review & editing. YS: Data curation, Formal analysis, Investigation, Project administration, Writing – review & editing. JS: Formal analysis, Supervision, Validation, Writing – review & editing. HY: Data curation, Validation, Writing – review & editing. YZ: Conceptualization, Data curation, Formal analysis, Investigation, Writing – review & editing. ZS: Formal analysis, Methodology, Software, Writing – review & editing. LQ: Formal analysis, Methodology, Software, Writing – review & editing. WZ: Software, Writing – review & editing, Formal analysis, Investigation, Methodology. XW: Conceptualization, Formal analysis, Funding acquisition, Project administration, Resources, Writing – review & editing.

## Footnotes

^1^ www.clinicaltrials.org

## Glossary

AST/ALT ratio: aspartate aminotransferase/alanine aminotransferase ratio
INR: international standard ratio
CLD: chronic liver disease
AD: acute decompensation
EASL: European Association for the Study of the Liver
CLIF: Chronic Liver Failure
CANONIC: Consortium Acute-on-Chronic Liver Failure in Cirrhosis
AST: aspartate aminotransferase
ALT: alanine aminotransferase
TBIL: total bilirubin
GGT: gamma-glutamyl transpeptidase
ALB: albumin
AKP: alkaline phosphatase
Scr: serum creatine
WBC: white blood cell count
NC: neutrophil count
LC: lymphocyte count
HGB: hemoglobin
PLT: platelet
HE: hepatic encephalopathy
HRS: hepatorenal syndrome
MELD: model for end-stage liver diseases
ACLF: acute-on-chronic liver failure
CLIF-C ACLF: chronic liver failure consortium ACLF
CLIF-C AD: chronic liver failure consortium acute decompensations
IQR: interquartile range
FIO2: fraction of inspiration O2
MAP: mean arterial pressure
CI: 95% confidence interval
HBV: hepatitis B virus
BUN: blood urea nitrogen
HBV: Hepatitis B virus
INR: international normalized ratio
