# Supplementary material for: Association of AST/ALT ratio with 90-day outcomes in patients with acute exacerbation of chronic liver disease: a prospective multicenter cohort study in China
Source: Front Med (Lausanne). 2024 Mar 21;11:1307901. doi: 10.3389/fmed.2024.1307901 (PMC10993385; doi:10.3389/fmed.2024.1307901)
Supplement: Supplementary file 1 [file Table_1.DOCX]

| **Table S1. Comparison of the characteristics of the patients with cirrhosis according to the aspartate aminotransferase/alanine aminotransferase ratio** | | | | | |
| --- | --- | --- | --- | --- | --- |
| **Characteristics** | **ASL/ALT Ratio(N=2803)** | | | | ***P* value** |
|  | **≤1.38** |  | **＞1.38** |  |  |
|  | N=1496 |  | N=1307 |  |  |
| **Demographics** |  |  |  |  |  |
| Age, (mean±SD) | 49.8±0.3 |  | 53.1±0.3 |  | <0.001 |
| Gender (male), No. (%) | 1162 (77.7) |  | 882 (67.5) |  | <0.001 |
| **Etiology, No. (%)** |  |  |  |  |  |
| HBV | 1063 (71.1) |  | 702 (53.7) |  | <0.001 |
| Alcoholic | 100 (6.7) |  | 209 (16.0) |  | <0.001 |
| Others | 333(22.3) |  | 396 (30.3) |  | <0.001 |
| **Complications, No. (%)** |  |  |  |  |  |
| Ascites | 840(56.1) |  | 875 (66.9) |  | <0.001 |
| Gastrointestinal bleeding | 307(20.5) |  | 269 (20.6) |  | 0.969 |
| Bacterial Infection | 359(24.0) |  | 364 (27.9) |  | 0.020 |
| Hepatic encephalopathy |  |  |  |  |  |
| Not overt | 1363 (91.1) |  | 1143 (87.5) |  | <0.001 |
| Grade1 | 46 (3.1) |  | 64 (4.9) |  | 0.080 |
| Grade2 | 57 (3.8) |  | 67 (5.1) |  | 0.091 |
| Grade3 | 20 (1.3) |  | 27 (2.1) |  | 0.176 |
| Grade4 | 10 (0.7) |  | 6 (0.5) |  | 0.463 |
| **Laboratory results （mean±SD）** |  |  |  |  |  |
| Alanine aminotransferase, IU/L | 282.6 ±11.3 |  | 66.2±3.7 |  | <0.001 |
| Aspartate aminotransferase, IU/L | 210.1±8.3 |  | 135.6±7.2 |  | <0.001 |
| Total bilirubin, mg/dL | 9.7±0.3 |  | 8.5±0.3 |  | 0.003 |
| International normalized ratio | 1.7±0.02 |  | 1.7±0.02 |  | 0.910 |
| Creatinine, mg/dL | 0.8±0.01 |  | 0.9±0.02 |  | 0.015 |
| Blood urea nitrogen, mmol/dL | 6.1±0.1 |  | 7.6±0.4 |  | <0.001 |
| Albumin, g/L | 32.0±0.2 |  | 29.7±0.2 |  | <0.001 |
| White blood cell, 10^9^/L | 5.7±0.1 |  | 5.9±0.1 |  | 0.282 |
| Platelet, 10^9^/L | 92.2±1.5 |  | 91.3±2.3 |  | 0.722 |
| Hemoglobin, g/L | 113.1±0.7 |  | 99.2±0.7 |  | <0.001 |
| Sodium, mmol/L | 137.7±0.1 |  | 136.5±0.2 |  | <0.001 |
| **Score（mean±SD）** |  |  |  |  |  |
| MELD | 17.6±0.2 |  | 17.1±0.2 |  | 0.243 |
| MELD-Na | 18.6±0.2 |  | 18.8±0.2 |  | 0.491 |
| CTP | 8.9±0.1 |  | 9.5±0.1 |  | <0.001 |
| **28-day adverse outcome, No. (%)** |  |  |  |  |  |
| Death | 128(8.6) |  | 114 (8.7) |  | 0.876 |
| Transplantation | 66 (4.4) |  | 91(7.0) |  | 0.003 |
| **90-day adverse outcome, No. (%)** |  |  |  |  |  |
| Death | 210(14.0) |  | 220 (16.8) |  | 0.041 |
| Transplantation | 92 (6.1) |  | 119(9.1) |  | 0.003 |
| Abbreviations: SD, standard deviation; HBV, hepatitis B virus; IQR, interquartile range; MELD, the model of end-stage liver disease; MELD-Na, the model of end-stage liver disease with sodium; CTP, Child-Turcotte-Pugh | | | | | |
