# Supplementary figures and images for: Association of AST/ALT ratio with 90-day outcomes in patients with acute exacerbation of chronic liver disease: a prospective multicenter cohort study in China
Source: Front Med (Lausanne). 2024 Mar 21;11:1307901. doi: 10.3389/fmed.2024.1307901 (PMC10993385; doi:10.3389/fmed.2024.1307901)

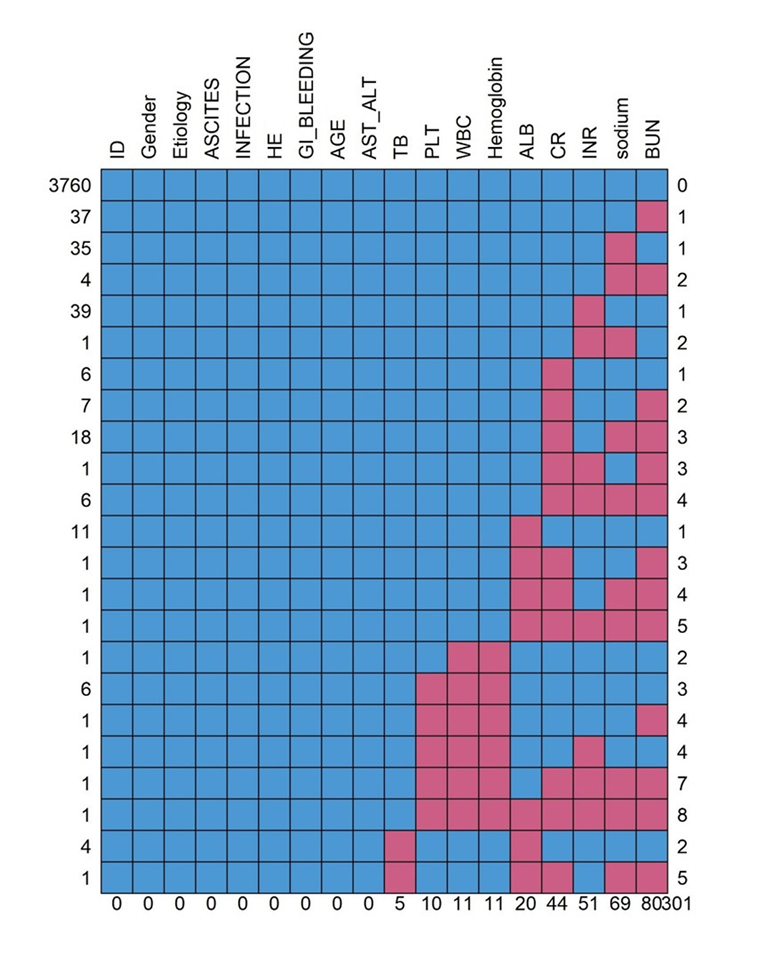

Supplement: Supplementary file 2 [file Image_1.TIF]

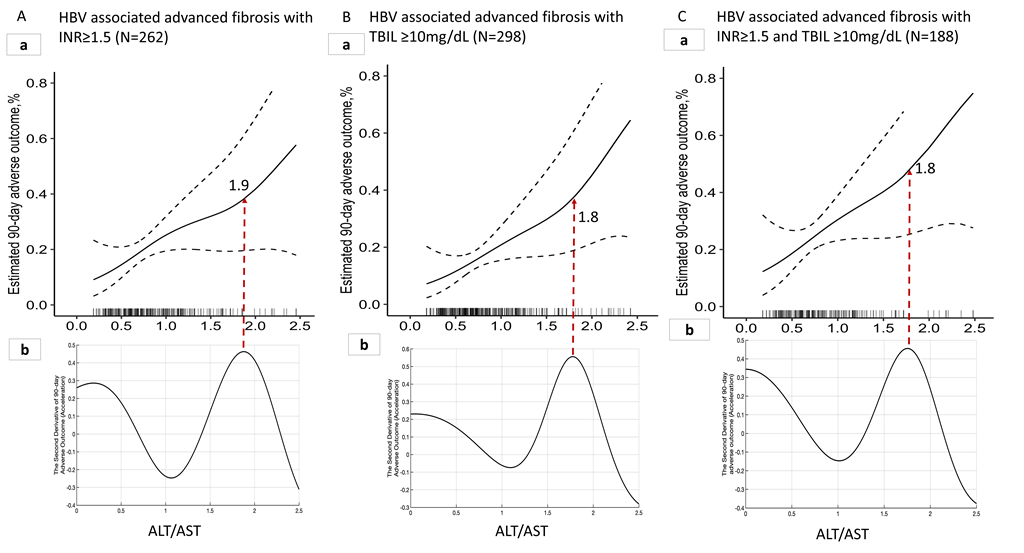

Supplement: Supplementary file 3 [file Image_2.TIF]

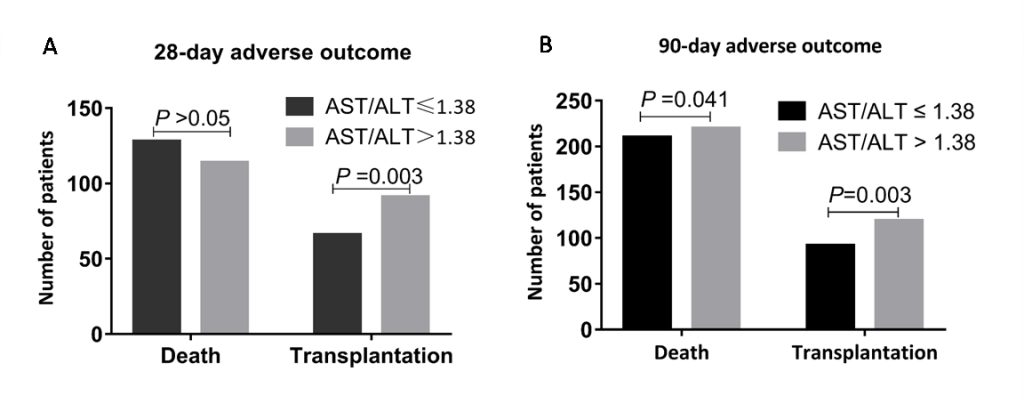

Supplement: Supplementary file 4 [file Image_3.tif]
